# In light of breathing: environmental light is an important modulator of breathing with clinical implications

**DOI:** 10.3389/fnins.2023.1217799

**Published:** 2023-07-13

**Authors:** Aaron A. Jones, Deanna M. Arble

**Affiliations:** Department of Biological Sciences, Marquette University, Milwaukee, WI, United States

**Keywords:** light, breathing, circadian rhythms, retinal ganglion cell, melanopsin, Brn3b

## Abstract

In vertebrate animals, the automatic, rhythmic pattern of breathing is a highly regulated process that can be modulated by various behavioral and physiological factors such as metabolism, sleep–wake state, activity level, and endocrine signaling. Environmental light influences many of these modulating factors both indirectly by organizing daily and seasonal rhythms of behavior and directly through acute changes in neural signaling. While several observations from rodent and human studies suggest that environmental light affects breathing, few have systematically evaluated the underlying mechanisms and clinical relevance of environmental light on the regulation of respiratory behavior. Here, we provide new evidence and discuss the potential neurobiological mechanisms by which light modulates breathing. We conclude that environmental light should be considered, from bench to bedside, as a clinically relevant modulator of respiratory health and disease.

## Introduction

In vertebrate animals, breathing is a crucial biological function that is modulated by complex interactions between various behavioral and physiological factors. At the level of the brain, pontomedullary regions in the brainstem control the automatic, rhythmic pattern of breathing (reviewed by Smith, 2022). Respiratory pattern generation within the brainstem can be modulated by chemical signals such as blood gas levels (Nattie, 1999), and neuroendocrine signaling (Behan and Kinkead, 2011; Gauda et al., 2020), or via mechanical feedback by stretch receptors and proprioceptors (von Euler, 1973; Yu, 2022). Additionally, higher order midbrain and forebrain regions provide descending input to the neural respiratory network to modify the respiratory pattern in response to changes in metabolic, sleep–wake, vigilance, and emotional states (reviewed by Schottelkotte and Crone, 2022).

Light is one of the most powerful environmental regulators of behavior. In addition to visual processing, light is the primary synchronizer for the endogenous circadian clock which shapes 24-h rhythms in physiology and behavior. Furthermore, light exerts direct, non-circadian effects on certain biological processes such as sleep–wake, alertness, body temperature, the pupillary light reflex, and mood (Chen et al., 2011; Hubbard et al., 2013; Fernandez et al., 2018; Rupp et al., 2019). However, less attention has been given to light’s effects on respiratory behavior. Mounting evidence implicates a prominent role for environmental light in modulating breathing via higher order midbrain and hypothalamic structures. Here, we discuss light’s known effects on breathing and their potential mechanisms.

## Light modulates breathing

**Figure 1:**
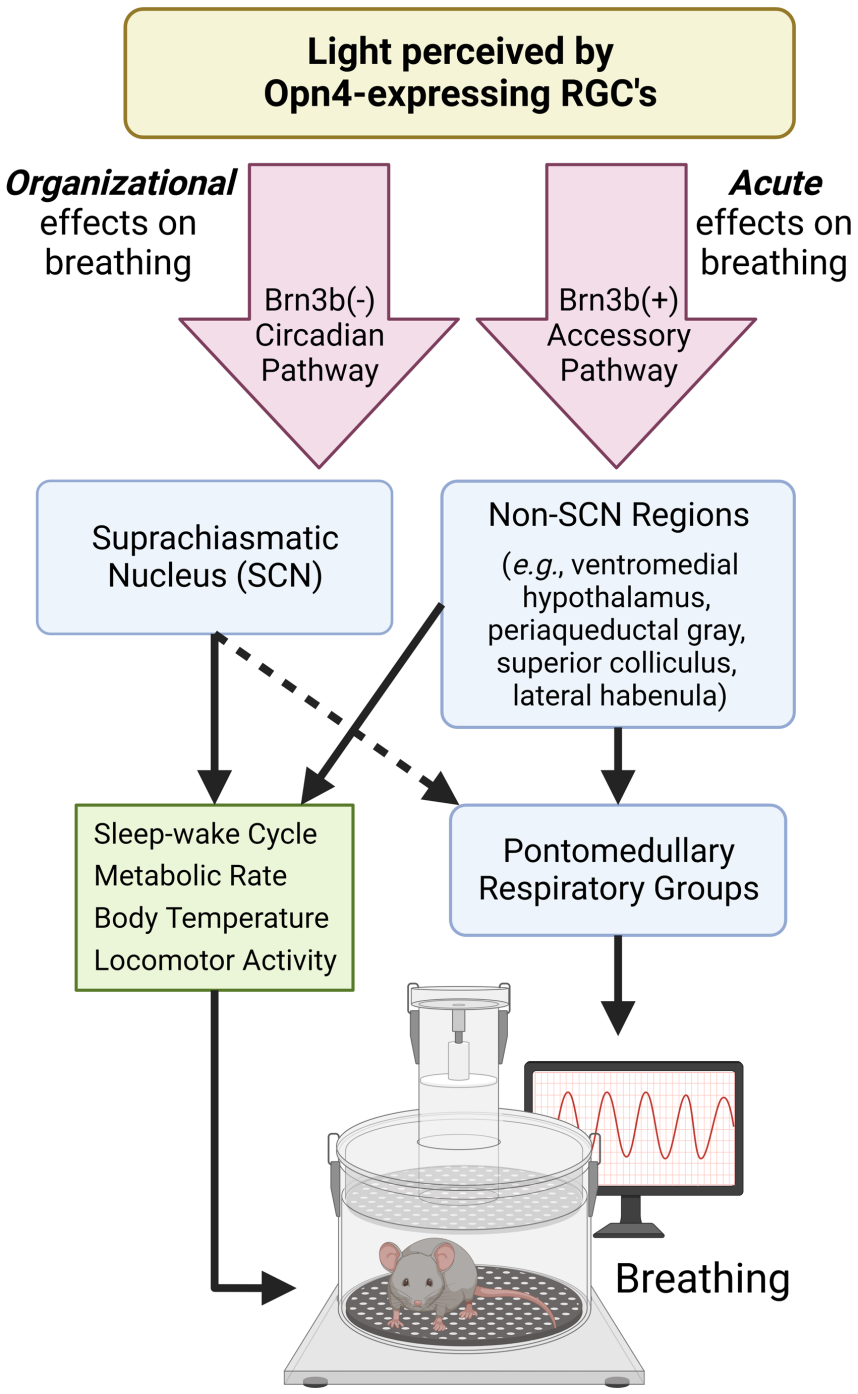
Proposed pathways by which light modulates breathing. Opn4-expressing retinal ganglion cells (RGCs) detect environmental light to mediate non-image forming processes. Opn4-expressing RGCs that do not express Brn3b project to the SCN and aid in the organization and synchronization of circadian rhythms. Many SCN-controlled variables (green box) can also influence breathing. Opn4-expressing RGCs that express Brn3b project to many hypothalamic and midbrain regions known to regulate breathing and breathing-related behaviors. The dotted arrow indicates connections that are not well-characterized. SCN, suprachiasmatic nucleus; RGC, retinal ganglion cell. Created with Biorender.com.

Light has the ability to modulate breathing across multiple timescales and for seemingly different biological purposes. In this section, we separately categorize the daily and seasonal effects of light as “organizational” and the distinctly different, immediate effects of light as “acute” (Figure 1).

### Light organizes daily and seasonal patterns of breathing

While daily rhythms refer to any rhythm that occurs on a 24-h basis, circadian rhythms in physiology and behavior are orchestrated endogenously by the suprachiasmatic nucleus (SCN), the master circadian clock located in the anterior hypothalamus. The SCN contains its own endogenous pacemaker with an approximate 24-h period and is synchronized by environmental light. Indeed, light is the dominant *Zeitgeber* or “time-giver” for the SCN, allowing for precise matching of the SCN to the external, 24-h solar day. Through direct, autonomic, and endocrine signaling, the SCN subsequently organizes circadian rhythms in physiology and behavior (Rosenwasser and Turek, 2015), including locomotor activity, sleep–wake cycles, and metabolism. Most animals studied to date also exhibit a daily rhythm in breathing. For example, minute ventilation, the product of respiratory rate and tidal volume, exhibits a daily rhythm that peaks during the active phase in rodents (Stephenson et al., 2001; Seifert and Mortola, 2002; Purnell and Buchanan, 2020; Jones et al., 2021) and humans (Bosco et al., 2003; Adamczyk et al., 2008). The daily rhythm in breathing can be phase shifted by acute perturbation of the light:dark cycle (Mortola, 2007) and, in some instances (Spengler et al., 2000; Jones et al., 2021), but not all (Bosco et al., 2003), may be weakened by an absence of light cues. Thus, the daily rhythm in breathing is fundamentally organized by the external light environment.

The organizational effects of light on breathing have clinical implications and are apparent in some instances of respiratory disease, such as sleep apnea. For example, the apnea-hypopnea index (AHI), key to the diagnosis of sleep apnea, exhibits a daily rhythm in a rodent model of sleep apnea (Fink et al., 2014) and humans with sleep apnea (Butler et al., 2015). When individuals with obstructive sleep apnea slept at all phases of the circadian cycle, AHI was found to be higher during the individual’s biological day, while the number of oxygen desaturation events was higher during the individual’s biological night (Butler et al., 2015). Moreover, in shift workers with obstructive sleep apnea, AHI and apnea duration were increased when individuals slept during the day following a night shift as compared to those that slept during the night following a day shift (Laudencka et al., 2007). These studies suggest that circadian biology contributes to respiratory pathophysiology and that circadian disruption and/or misalignment with the external light environment may exacerbate respiratory symptoms. While light may have clinical implications for other respiratory diseases with time-specific symptomatology, including sudden unexpected death in epilepsy, chronic asthma, obstructive pulmonary disease, and COVID-19 (Mortola, 2004; Maidstone et al., 2021; Purnell et al., 2021; van Goor et al., 2022), few studies have explored this hypothesis.

In addition to daily regulation, light may drive seasonal changes in breathing as well. Light duration/availability changes seasonally in many locations of the world, which can alter various aspects of circadian physiology (Coomans et al., 2015). It was recently demonstrated in C57Bl/6 J mice that altering light duration to mimic seasonal changes in light availability while preserving the endogenous circadian period leads to changes in the respiratory pattern across the day and weakens the relationship between ventilation and metabolism in a time-dependent manner (Jones et al., 2021). Indeed, mice housed under short days with only 5 h of light demonstrate a daily rhythm in minute ventilation that is controlled by tidal volume rather than the normally prevailing respiratory rate. Additionally, time-dependent differences were observed under the short day after normalizing ventilation to metabolic rate, suggesting some dissociation of minute ventilation and metabolism across the day. In turn, mice housed on longer days with 19 h of light demonstrate a non-detectable rhythm in minute ventilation due to more variable breathing during the light phase (Jones et al., 2021). These findings point to a role of seasonal light duration in reorganizing daily respiratory patterns. In humans, sleep-disordered breathing is worse in individuals with obstructive sleep apnea during the winter months (Cassol et al., 2012), which may correlate with a decrease in light availability. However, additional studies are needed to understand the relationship between seasonal light duration and breathing, especially in the context of respiratory disease.

### Light acutely modulates physiology to elicit immediate changes in breathing

While light is an important organizational cue for biological rhythms, light also has direct effects on physiology and behavior separate from the circadian clock. For example, light can directly elicit an acute suppression of locomotor activity, body temperature, and wakefulness (Hatori et al., 2008; Rupp et al., 2019) and acutely alter arousal/alertness (Lok et al., 2018). Notably, all these variables may influence respiratory physiology. Mice assessed using plethysmography in lit conditions, for example, demonstrate decreased ventilation compared to mice assessed in dark conditions, even when controlling for circadian phase (Quindry et al., 2016). While this indicates that light can directly suppress breathing in nocturnal rodents, the particular neural mechanism by which light acutely regulates breathing is poorly understood.

Apnea pathophysiology also appears to be acutely affected by light independent of circadian timing. When healthy individuals sleep in brightly-lit rooms, their breathing becomes more irregular, and they exhibit apneic events that are not otherwise observed when sleeping in dark rooms (Yamauchi et al., 2014). The investigators attribute the increased breathing irregularity to an augmented sympathetic tone, as evidenced by a concurrent increase in the low-frequency/high-frequency power ratio. Therefore, light may exert its direct effects on breathing via acute changes in autonomic function. This notion agrees with the findings that acute light exposure enhances muscle sympathetic nerve activity (Saito et al., 1996) and suppresses vagal parasympathetic activity (Yuda et al., 2016) in humans. These albeit limited observations point to a direct pathway by which light affects respiratory disease, independent of circadian timing.

## Potential mechanisms by which light modulates breathing

Retinal ganglion cells (RGCs) that express the photopigment melanopsin (Opn4) detect environmental light (Panda et al., 2002) and make numerous central projections that result in the modulation of hormone release, activity levels, and mood over both daily and seasonal timescales (LeGates et al., 2014; Leclercq et al., 2021). Responsible for non-image forming processes, Opn4-expressing RGCs are intrinsically responsive to light and send projections to the SCN to synchronize the master clock to the external light environment (LeGates et al., 2014). Additionally, Opn4-expressing RGCs project to many non-SCN targets (Li and Schmidt, 2018). The projections to non-SCN regions, sometimes referred to as the Opn4-expressing “accessory pathway,” mediate light’s direct, acute effects on a variety of biological processes including body temperature, sleep–wake state, and mood (Fernandez et al., 2018; Rupp et al., 2019). In the present article, we demonstrate that breathing is also acutely modulated by this non-SCN, accessory pathway.

### Light organizes daily breathing by providing timing cues to the circadian clock

The organizational effects of light on daily breathing rhythms likely stem from light’s ability to synchronize the SCN. The SCN, in turn, may influence daily respiratory patterns either directly via neuronal outputs and/or indirectly by organizing behavioral rhythms (e.g., activity, sleep/wake, metabolic rate) that subsequently affect daily breathing. A recent lesion study gives some insight into this hypothesis. Following an SCN lesion, investigators found that the animals’ rhythms in respiratory rate and minute ventilation were abolished when held in constant darkness (Purnell and Buchanan, 2020). Given that a SCN lesion is known to also eliminate day-night differences in locomotor activity and metabolic rate (Coomans et al., 2013), as well as rhythms in sleep–wake and body temperature (Ibuka et al., 1980; Eastman et al., 1984), this finding alone cannot ascertain whether the organizational effects of the SCN on breathing occur via direct neuronal connections or through the indirect organization of key physiological variables that subsequently influence breathing. However, previous studies suggest that the SCN may affect breathing rhythms independently of its ability to organize other variables related to breathing. For example, an observable daily rhythm in breathing persists even when controlling for sleep–wake state (Stephenson et al., 2001) and activity level (Seifert and Mortola, 2002). Additionally, rhythms in body temperature and metabolic rate can be phase-uncoupled from breathing under situations of light perturbation (Spengler et al., 2000; Mortola, 2007; Jones et al., 2021). Therefore, SCN-dictated behaviors alone seem insufficient to fully organize the daily rhythm in breathing.

It is likely that modulation of the respiratory system itself via the SCN at least partially contributes to the organization of daily breathing rhythms. The SCN is responsible for the synchronization of rhythmic clock gene expression which occurs in nearly every cell studied to date. Indeed, ~24-h rhythmic translation of clock proteins are observed throughout the respiratory system including the caudal brainstem, cervical spinal cord, diaphragm, lungs, and airway (Bando et al., 2007; Hadden et al., 2012; Kelly et al., 2020). Additionally, known genes related to respiratory function also oscillate in these structures including those involved in phrenic motor plasticity, airway contraction, and mucin secretion (Bando et al., 2007; Kelly et al., 2020). The SCN appears to entrain airway tissues via the vagal nerve (Bando et al., 2007), however, it is currently unknown how the SCN confers timing to other respiratory structures. While it is unclear whether these other mechanisms contribute to daily breathing behavior, we recently demonstrated that eliminating molecular time-keeping specifically within respiratory cells of the autonomic nervous system abolishes circadian variation in the hypoxic ventilatory response (Jones et al., 2023). This finding suggests that the circadian clock may organize daily breathing rhythms, in part, via the organization of clock genes in the neural respiratory network.

With respect to seasonal changes in resting breathing (Jones et al., 2021) and respiratory pathophysiology (Cassol et al., 2012), the underlying neurobiological mechanisms remain unclear. Animal studies suggest a possible role of serotonin (5HT) signaling. Exposing mice and chipmunks to summer-like days with longer light duration increases 5HT levels in the dorsal raphe nucleus (DRN) and increases the excitability and firing rate of DRN neurons (Goda et al., 2015; Green et al., 2015). The DRN has the potential to modulate breathing by providing input to respiratory motor neurons of the upper airway (Guo et al., 2020) or through hetero-exchange of 5HT to medullary serotonergic neurons of the central respiratory network (Wu et al., 2019). The role of seasonal light availability in modulating breathing via 5HT signaling is worthy of further investigation.

Collectively, the external light environment organizes daily ventilatory rhythms through its synchronization of the SCN. While the underlying mechanisms by which the circadian clock facilitates this is unclear, it appears likely that the SCN organizes breathing rhythms both directly via the synchronization of cellular rhythms within respiratory structures and indirectly through its organization of behavioral and physiological rhythms closely-related to breathing.

### Light acutely modulates breathing by activating non-SCN neuronal pathways

Light’s ability to organize breathing rhythms via the SCN (as discussed in the preceding section) may occur in parallel to its ability to directly and acutely modulate breathing on much faster timescales. In animals without a functioning molecular clock, for example, a light:dark cycle is sufficient to maintain certain behavioral rhythms such as locomotor activity and metabolic rate (Adamovich et al., 2019). This indicates that light has the ability to immediately drive behavior independently of the SCN (also referred to as “light masking”). In line with this notion, we recently identified that mice lacking a functional molecular clock continue to exhibit a detectable daily rhythm in minute ventilation when housed on a standard light:dark cycle (Jones et al., 2023). This suggests that, like some other behaviors, light can synchronize daily breathing rhythms independent of the circadian clock. In humans, the daily ventilation rhythm appears partially disrupted under constant dim light (Spengler et al., 2000), suggesting that transitions from light to dark and vice versa may be important signals in stabilizing ventilatory rhythms independent of the circadian clock. However, since subjects were placed on bed rest and kept awake for 41 h, an alternative interpretation is that decreased/non-rhythmic activity levels and/or decreased alertness due to mild sleep deprivation may account for disruption of the daily rhythm in ventilation and not absence of light cues *per se*.

While Opn4-expressing RGCs directly innervate the SCN, they also project to numerous brain regions other than the SCN (Li and Schmidt, 2018). Chemogenetic activation of all Opn4-expressing RGCs excites numerous thalamic, hypothalamic, and limbic brain regions that promote an alert vigilance state (Milosavljevic et al., 2016). Several of these hypothalamic regions are involved in respiratory control, namely the dorsomedial hypothalamus and paraventricular nucleus which have secondary and primary connections to the respiratory pattern generator, respectively (Mack et al., 2007; Fukushi et al., 2019). These regions are positioned to contribute to light’s direct effects on breathing, and may partially explain the interaction between light, vigilance state, and breathing. Opn4-expressing RGCs can be further sub-divided by the expression of the transcriptional marker Brn3b. Brn3b-expressing RGCs comprise the “accessory” pathway as they do not project to the SCN and can drive biological processes independently of the SCN, such as the pupillary light reflex (Chen et al., 2011) and mood regulation (Fernandez et al., 2018). This is in contrast to RGCs that do not express Brn3b which primarily project to the SCN (Li and Schmidt, 2018).

**Figure 2:**
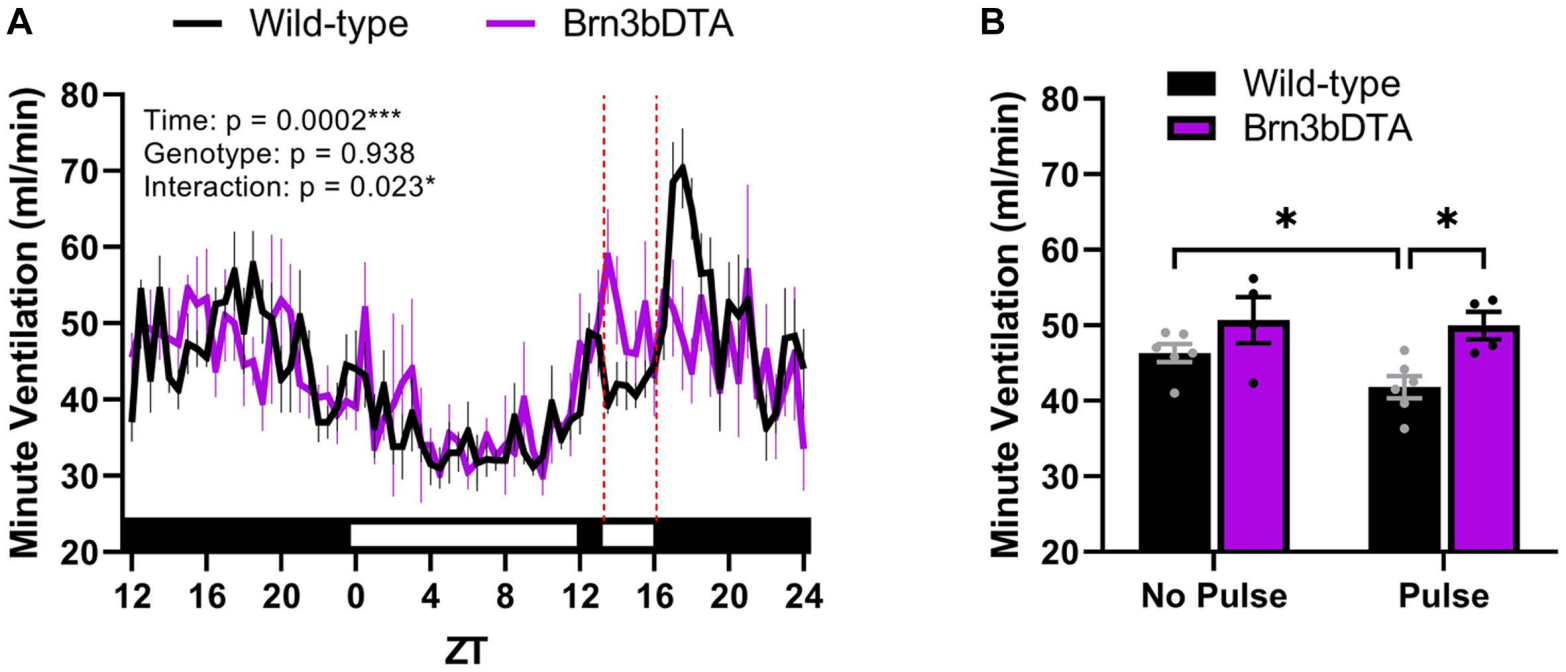
Light acutely suppresses breathing via Brn3b-expressing retinal ganglion cells. **(A)** Minute ventilation of wild-type mice (*n* = 6) and mice with diphtheria toxin A-mediated ablation of Brn3b-expressing retinal ganglion cells (*n* = 4) during a 3-h light pulse early in the dark phase. In wild-type mice only, breathing decreases in response to acute light exposure and rebounds after its removal. Red dotted lines denote the time at which the light pulse occurred. **(B)** Mean minute ventilation of wild-type and Brn3bDTA mice during the 3-h light pulse. Wild-type mice demonstrate a significant reduction in minute ventilation during the light pulse (relative to the same ZT range from the night prior), while mice lacking Brn3b-expressing cells do not. **(A,B)** Repeated measures two-way ANOVA with Sidak’s post-hoc, ***p < 0.001, *p < 0.05. All data are expressed as the mean ± SEM. Methods: Male and female Brn3bDTA (Opn4^cre/+^; Brn3b^DTA/+^) and wild-type littermates (Opn4^cre/+^; Brn3b^+/+^) were bred in-house from founders provided by Dr. Tiffany Schmidt (Northwestern University) according to the breeding strategy of prior studies (Chen et al., 2011; Fernandez et al., 2018). Minute ventilation was measured continuously for 37 h at 11–24 weeks of age by means of whole-body plethysmography (SCIREQ/Emka Technologies, Paris, France) using previously described parameters (Jones et al., 2023). All methods were reviewed, approved by, and performed according to the guidelines of the Institutional Animal Care and Use Committee of Marquette University (Milwaukee, WI). WT, wild-type; DTA, diphtheria toxin A; ZT, zeitgeber time. [*Original data*].

Recently, we identified that the accessory pathway is also responsible for driving acute effects on breathing. Indeed, compared to wild-type littermates, mice lacking Brn3b-expressing RGCs fail to exhibit an acute suppression of minute ventilation when a 3-h light pulse is delivered during the early dark phase (Figures 2A,B). This finding is similar to a prior study demonstrating that Brn3b-expressing RGCs mediate light’s acute effects on body temperature and sleep (Rupp et al., 2019). Moreover, we found that the breathing of wild-type mice exhibited a pronounced rebound following light removal (Figures 2A), which may be due to an exaggerated increase in locomotor activity following acute negative masking. One interpretation is that the acute light-mediated reduction in breathing is secondary to decreased body temperature or wakefulness. Nonetheless, Brn3b-expressing RGCs directly project to numerous regions known to be involved in breathing (Hattar et al., 2006; Li and Schmidt, 2018), offering alternative routes by which light may alter breathing independent of the SCN, body temperature, or sleep/activity states.

Brn3b-expressing RGCs may modulate breathing via several target regions including: *the ventromedial hypothalamus* which projects to catecholaminergic neurons of the brainstem to facilitate sympathetic respiratory responses (Lindberg et al., 2013); *the midbrain periaqueductal gray* which modulates breathing in response to noxious stimuli (Flak et al., 2017) and optimizes eupneic breathing in response to sensory information under survival situations (Subramanian and Holstege, 2010); *the superior colliculus*, a region that increases breathing rate when stimulated (Keay et al., 1988) and projects to neurons of the respiratory pattern generator (Yang et al., 2020); and *the lateral habenula*, which induces apnea-like breathing through its inhibition of the raphe nuclei (Wang et al., 2014). While all of these regions are implicated in respiratory control, it remains unclear to what extent Brn3b-expressing RGCs influence breathing via photic modulation of these regions. Nonetheless, based on the present data, it is evident that light activates non-SCN neuronal pathways to acutely regulate breathing. Additional studies are warranted to further define the relative contributions of non-SCN retinal pathways in acutely modifying respiratory behavior.

## Conclusions and future directions

This perspective highlights the important role of environmental light in shaping respiratory behavior. Light can acutely influence various aspects of breathing and organize breathing across daily and seasonal time scales which has implications for human health. While intrinsically-photosensitive retinal circuitry to SCN and non-SCN neural populations contribute to these effects (Figure 1), it is unclear to what extent the ventilatory effects of light are secondary to other known influencers of breathing within the shared neuronal circuitry. Moving forward, we argue that several key questions should be addressed: What features of light exposure are the most potent modulators of breathing (e.g., light duration, timing, intensity, and wavelength)? Which neuronal connections/mechanisms predominantly mediate light’s effects on breathing? In what ways should lighting be incorporated into the design and execution of respiratory studies? To what extent does aberrant or mistimed light exposure influence respiratory pathology? The conceptual framework outlined in this article indicates a compelling need to consider these effects when conducting rodent and human respiratory studies.

## Data availability statement

The original contributions presented in the study are included in the article/supplementary material, further inquiries can be directed to the corresponding author.

## Ethics statement

The animal study was reviewed and approved by the Institutional Animal Care and Use Committee of Marquette University.

## Author contributions

AJ collected, graphed, analyzed, and interpreted all data. AJ and DA conceived or designed the experiment. AJ and DA wrote and edited the manuscript. All authors contributed to the article and approved the submitted version.

## Funding

This study was supported by Marquette University funds. AJ is supported by the Marquette University Richard W. Jobling Distinguished Research Assistantship.

## Conflict of interest

The authors declare that the research was conducted in the absence of any commercial or financial relationships that could be construed as a potential conflict of interest.

## Publisher’s note

All claims expressed in this article are solely those of the authors and do not necessarily represent those of their affiliated organizations, or those of the publisher, the editors and the reviewers. Any product that may be evaluated in this article, or claim that may be made by its manufacturer, is not guaranteed or endorsed by the publisher.
